# Predicting Plasma Concentrations of Bisphenol A in Children Younger Than 2 Years of Age after Typical Feeding Schedules, using a Physiologically Based Toxicokinetic Model

**DOI:** 10.1289/ehp.0800073

**Published:** 2008-11-14

**Authors:** Andrea N. Edginton, Len Ritter

**Affiliations:** 1 School of Pharmacy, University of Waterloo, Waterloo, Ontario, Canada;; 2 Department of Environmental Biology, University of Guelph, Guelph, Ontario, Canada

**Keywords:** bisphenol A, ontogeny, physiologically based toxicokinetics

## Abstract

**Background:**

Concerns have recently been raised regarding the safety of potential human exposure to bisphenol A (BPA), an industrial chemical found in some polycarbonate plastics and epoxy resins. Of particular interest is the exposure of young children to BPA via food stored in BPA-containing packaging.

**Objectives:**

In this study we assessed the age dependence of the toxicokinetics of BPA and its glucuronidated metabolite, BPA-Glu, using a coupled BPA–BPA-Glu physiologically based toxicokinetic (PBTK) model.

**Methods:**

Using information gathered from toxicokinetic studies in adults, we built a PBTK model. We then scaled the model to children < 2 years of age based on the age dependence of physiologic parameters relevant for absorption, distribution, metabolism, and excretion.

**Results:**

We estimated the average steady-state BPA plasma concentration in newborns to be 11 times greater than that in adults when given the same weight-normalized dose. Because of the rapid development of the glucuronidation process, this ratio dropped to 2 by 3 months of age. Simulation of typical feeding exposures, as estimated by regulatory authorities, showed a 5-fold greater steady-state BPA plasma concentration in 3- and 6-month-olds compared with adults, reflecting both a reduced capacity for BPA metabolism and a greater weight-normalized BPA exposure. Because of uncertainty in defining the hepatic BPA intrinsic clearance in adults, these values represent preliminary estimates.

**Conclusions:**

Simulations of the differential BPA dosimetry between adults and young children point to the need for more sensitive analytical methods for BPA to define, with greater certainty, the adult hepatic BPA intrinsic clearance, as well as a need for external exposure data in young children.

Bisphenol A (BPA) is an industrial chemical found in polycarbonate plastics and epoxy resins such as food-can linings, bottles, and dental fillings. The weak estrogenic activity of BPA (Laws et al. 2000; Stoker et al. 1999) has raised questions as to its safety in humans. Human exposure to BPA is potentially widespread, and young children particularly are seen by regulatory authorities as a population of special concern [National Toxicology Program (NTP) 2008]. Their susceptibility is based on their reduced capacity to eliminate xenobiotics in general (Hines 2008) as well as their estimated higher weight-normalized daily exposure to BPA compared with adults [European Food Safety Authorities (EFSA) 2006; NTP 2008]. Having a method to estimate the age dependence of internal BPA exposure is critical for developing a reasonable assessment of BPA risk to children.

In response to the identification of young children as a higher risk population, we investigated the exposure of children to BPA using physiologically based toxico kinetic (PBTK) modeling. PBTK models have been widely used in human health risk assessment to assess the potential internal human exposure to environmental compounds in the absence of direct human toxicokinetic data (Andersen 2003; Bruckner et al. 2004). Typically, toxicokinetic data from animals are used to build a compound-specific PBTK model that is then parameterized with information for humans (e.g., blood flows, organ volumes) (Bruckner et al. 2004; Young et al. 2001). Although interspecies scaling represents the primary use of PBTK models, intraspecies scaling also represents a valuable means for assessing exposures in subpopulations (Edginton et al. 2008). Pharmaco-/ toxicokinetic scaling for the purposes of both human health risk assessment and pharmaceutical drug development has focused on the pediatric subpopulation where physiologically based pharmacokinetic models are built for adult humans and scaled to children based on their physiologic differences (Bjorkman 2005; Edginton et al. 2006a, 2006b; Ginsberg et al. 2004; Yang et al. 2006).

BPA is completely and rapidly absorbed from the gastrointestinal tract (Volkel et al. 2002). Using an *ex vivo* method, Csanady et al. (2002) determined tissue:blood partition coefficients for human tissues after incubation with BPA. Because of the high lipophilicity of BPA, the adipose:blood partition coefficient (3.3) was two to three times greater than that of the other tested tissues (range, 0.9–1.82). Hepatic clearance of BPA to its glucuronidated metabolite, BPA-Glu, is rapid and complete, and BPA-Glu is the predominant substance found in plasma (Volkel et al. 2002). Urinary excretion is the only route of elimination for BPA-Glu (Volkel et al. 2002). The toxicokinetics of these substances have been studied only in adults. Toxicokinetic scaling to children using PBTK models remains the only means to reasonably assess relative internal BPA exposures.

The objectives of this modeling study were to estimate the differences in the average steady-state dose-normalized BPA plasma and urinary concentrations between adults and children < 2 years of age after BPA administration and to determine the expected average steady-state plasma concentrations of BPA and BPA-Glu in young children after typical feeding scenarios.

## Materials and Methods

### PBTK Model Structure and Software

**Figure 1:**
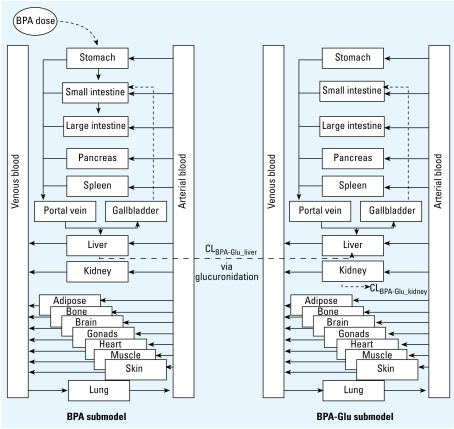
Schematic of the PBTK model structure consisting of BPA and BPA-Glu submodels. Input of BPA was to the stomach, thus simulating oral administration. Input of BPA-Glu was the hepatic metabolism of BPA to BPA-Glu in the liver.

In this study we used a nested coupled PBTK structure consisting of a BPA submodel coupled to a BPA-Glu submodel (Figure 1). We used PK-Sim software (version 4.0; Bayer Technology Services GmbH, Leverkusen, Germany) to generate each individual sub-model. Figure 1 graphically presents the PBTK model structure implemented in PK-Sim [presented by von Kleist and Huisinga 2007; Willmann et al. 2003a, 2005; described by differential and algebraic equations in the Supplemental Material (http://www.ehponline.org/members/2008/0800073/suppl.pdf)]. The model includes 15 organs as well as arterial, venous, and portal blood compartments. The organs are connected via blood flows, and the circulation system is closed via the lung. We coupled the sub-models on export to MoBi (version 2.0; Bayer Technology Services GmbH), a software package for the mechanistic and dynamic modeling of biologic processes and drug action. We linked the two submodels such that the hepatic clearance of BPA was the only source function for the BPA-Glu submodel, where the process was defined in the liver.

### PBTK Model Parameterization

#### BPA and BPA-Glu physicochemistry

Table 1 presents physicochemical and physiologic parameters of BPA and BPA-Glu.

#### Anatomical and physiologic parameters

We previously published the body weight, height, blood flows, and organ volumes for children and adults, as used in the PK-Sim software (Edginton et al. 2006b). Table 2 presents these parameter values as used in the pediatric and adult models for this study.

#### Absorption

The oral absorption model is that of Willmann et al. (2003b, 2004), and this model provided BPA input to the portal vein. We considered scaling the gastrointestinal parameters of gastrointestinal geometry, gastric emptying time, intestinal permeability, gastric and intestinal pH, small intestinal transit time, and intestinal surface area to children between 0 and 2 years of age. However, because of the high absorbed fraction (*f_a_* = 1) of orally administered BPA, these various inputs did not make a life-stage difference. As a result, all simulations used an *f_a_*value of 1.

#### Distribution

We used the algorithms of Rodgers and Rowland (2006) and Rodgers et al. (2005a, 2005b) to estimate tissue:plasma partition coefficients. Required input data were the fraction unbound in plasma, lipophilicity, and acid/base properties. Table 1 presents these input data for BPA and BPA-Glu. We slightly modified the Rodgers and Rowland (2006) and Rodgers et al. (2005a, 2005b) algorithm for children with respect to the interplay between neutral lipids and extracellular water. The volume fraction lipids in adipose tissue increases with age (Baker 1969) due to an adipocyte generation and cell volume growth (Boulton et al. 1978; Soriguer Escofet et al. 1996; Spalding et al. 2008). A relationship between the volume fraction lipids and interstitial space with age has been previously developed to adjust the adipose:plasma partition coefficient (Edginton et al. 2006b). We similarly altered the Rodgers and Rowland (2006) and Rodgers et al. (2005a, 2005b) algorithm through the interplay between the volume fraction neutral lipids and extracellular water for children. Permeability × surface area (PS) products define the rate of organ/tissue uptake and are estimated in PK-Sim. PS scaling to children used the following allometric function:


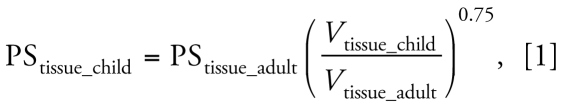


where *V* is the volume of the tissue.

#### Elimination

##### BPA clearance (CL_BPA_liver_)

We assumed that 100% of the elimination of BPA was attributable to metabolism to its glucuronidated metabolite, BPA-Glu. This is supported by Volkel et al. (2002), who determined that recovery of an isotope of BPA-Glu in plasma after human trials was equivalent to that of total BPA after addition of a glucuronidase. The enzyme responsible for this has not been directly assessed in humans. In rats, the responsible rat-specific isozyme is UDP-glucuronosyltransferase (UGT) 2B1 (UGT2B1) (Yokota et al. 1999). This rat-specific isozyme has a sequence and a substrate specificity similar to the human UGT2B7 isozyme. Using full-length rat UGT2B1 as a probe in a human cDNA library, one of two full-length clones was found to be UGT2B7 (Coffman et al. 1997), as characterized by Jin et al. (1993). Soars et al. (2003) demonstrated that dog UGT2B31, rat UGT2B1, and human UGT2B7 display similar substrate specificities, and that dog UGT2B31 had sequence alignments that were 75% and 73% identical to human UGT2B7 and rat UGT2B1. Using morphine, a known UGT2B7 substrate, as a probe, Pritchard et al. (1994) showed morphine glucuronidation at the 3-position by rat UGT2B1 with high velocity, a result confirmed by Soars et al. (2003) with the addition of minute observed amounts the 6-*O*-glucuronide. Coffman et al. (1997) have shown conclusively that human UGT2B7 can catalyze morphine glucuronidation at both the 3- and 6-positions, but the isozyme was approximately 10-fold more efficient at forming the 3-*O*-glucuronide. Another study examining the UGT responsible for diclofenac glucuronidation in humans found that rat UGT2B1 and human UGT2B7 both catalyzed this reaction with similar affinities (King et al. 2001). Based on the evidence of UGT2B1 and UGT2B7 sequence similarities and substrate overlaps, we considered UGT2B7 the enzyme responsible for BPA glucuronidation and thus used the enzyme ontogeny of UGT2B7 to scale the intrinsic clearance of BPA to BPA-Glu from adults to children. UGT2B7 activity in term neonates is only 5% that of adults, increases to 30% by 3 months of age, and reaches adult levels by 1 year of age (Edginton et al. 2006a).

We described the method of scaling clearance from adults to children (Edginton et al. 2006a). In brief, for hepatically cleared compounds, the plasma clearance (CL*_H_*; milliters per minute) from adults is converted to an intrinsic clearance (CL_int_; milliters per minute) using the well-stirred model (Equation 2) and physiologic information of liver blood flow (*Q_H_*; mL/min) and the fraction unbound in plasma (*f_u_*):


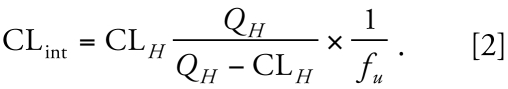


Clearance is then normalized to liver weight (milliters per minute/grams liver) and multiplied by the percentage of activity relevant to the age of the child to derive a liver weight–normalized intrinsic clearance for the child. Plasma clearances for children are derived by rearrangement of Equation 2 and by using the age-specific liver weight, blood flow, and estimated *f_u_* as derived using the method of McNamara and Alcorn (2002). This method adjusts the *f_u_* in adults to children based on the age dependence of albumin concentrations in plasma (McNamara and Alcorn 2002).

##### BPA-Glu clearance (CL_BPA-Glu_kidney_)

Urinary excretion is 100% responsible for BPA-Glu clearance in humans (Volkel et al. 2002). We optimized plasma CL_BPA-Glu_kidney_ during the development of the adult coupled model and compared this with values derived from Volkel et al. (2002). We also compared the estimated time course of BPA urinary excretion with that from Volkel et al. (2002). Allometric relationships as described by Hayton (2000) and modified slightly by Edginton et al. (2006a) formed the basis for scaling the age dependence of CL_BPA-Glu_kidney_.

We used the following equation to calculate the total BPA (BPA + BPA-Glu) average urinary concentration at steady state (*C*_BPA_urine_):


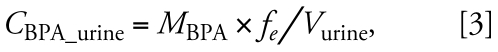


where *M*_BPA_ is the mass of BPA ingested per day, *f_e_* is the fraction excreted to urine, and *V*_urine_ is the volume of urine produced in 1 day. *f_e_* is 1 because 100% of the BPA dose is converted to BPA-Glu, and 100% of BPA-Glu is excreted to urine, results observed by Volkel et al. (2002). A linear interpolation of *V*_urine_, as taken from the International Commission on Radiological Protection (ICRP 2002), provided values for newborns and for 3-month-, 6-month-, 1-year-, 2-year-, and 30-year-olds of 300, 320, 350, 400, 425, and 1,600 (adult male) mL/day, respectively. Lakind and Naiman (2008) used this equation in a rearranged form to calculate the daily BPA exposure from urinary concentrations and daily urine volume.

### Development of the Adult BPA–BPA-Glu Coupled Model

We parameterized the BPA and BPA-Glu models in PK-Sim for an average 30-year-old male of 73 kg and 176 cm (ICRP 2002). We used the experimental plasma concentration time data of BPA-Glu after BPA administration from Volkel et al. (2002) to parameterize the unknown data of CL_BPA_liver_, BPA-Glu lipophilicity (used for calculation of tissue:plasma partition coefficients), and CL_BPA-Glu_kidney_. We simulated a BPA oral dose of 5 mg (Volkel et al. 2002). We set CL_BPA_liver_ for this male at the lowest integer that maintained the BPA concentrations lower than the 10-nM limit of detection from the Volkel et al. (2002) study. We then coupled the BPA and BPA-Glu submodels in MoBi to reflect the metabolism process. The change of intracellular concentration due to BPA metabolism to BPA-Glu (CL_BPA_liver_) is calculated as (CL_BPA_liver_ × *C*_cell_liver_BPA_ × *f*_u_BPA_/*K*_liver_BPA_)/*V*_cell_liver_, where *C*_cell_liver_BPA_ is the concentration of BPA in the liver intra-cellular space, *f_u_*__BPA_ is the unbound fraction of BPA in plasma, *K*_liver_BPA_ is the liver:plasma partition coefficient, and *V*_cell_liver_ is the intracellular volume of the liver [see Supplemental Material for full differential equations (http://www.ehponline.org/members/2008/0800073/suppl.pdf)].

The only unknown parameters at this point were the lipophilicity of BPA-Glu and CL_BPA-Glu_kidney_. We optimized these parameters using the plasma concentration–time data of BPA-Glu for all subjects studied by Volkel et al. (2002). The objective function was the root mean squared error measured as the root of the squared difference between the predicted and observed plasma concentrations for both compounds. For optimization, we used the Mobi-Toolbox for MatLab (Bayer Technology Services GmbH) and the MatLab [version 7 (R14); MathWorks Inc., Natick, MA, USA] fminsearch optimization routine without constraints. We used the optimized BPA-Glu lipophilicity in all simulations for children as a means to predict partition coefficients. We scaled the optimized adult CL_BPA-Glu_kidney_ to children as previously described. During the scaling procedure and simulations in children, we allowed no changes to the coupled model structure.

### Scaling the Adult Coupled Model to Children

Once we set the adult model of BPA and BPA-Glu, we made changes to scale the model to children. We used the following age-specific parameters: weight, height, organ volumes, blood flows, volume fraction of fat in adipose tissue, tissue:plasma partition coefficients, PS, CL_BPA_liver_, CL_BPA-Glu_kidney_, and *f_u_*.

### Simulations

First, we completed simulations after a BPA application of 1 μg/kg once per day to demonstrate the expected differences in the average dose-normalized plasma concentrations at steady state of BPA and BPA-Glu in adults and young children (0–2 years of age). Second, we used published environmental exposure data (EFSA 2006) (Table 3) to estimate the average plasma concentration at steady state in children and adults under typical feeding scenarios. Because EFSA (2006) exposure scenarios did not include breast-fed newborns, we included this group by calculating exposure based on average daily breast milk intake and total BPA concentration in breast milk. The average daily breast milk intake is 13 g/kg on the first day of life, increases gradually 98 g/kg on day 3, and reaches a relatively constant level of 155 g/kg from day 5 (Casey et al. 1986). In the early days, using the highest concentration of free plus conjugated BPA from Ye et al. (2008), which is 1.62 μg/L, 95% of which was free BPA, the newborn (postnatal age = 5 days; 3.5 kg) would receive 0.88 μg of BPA per day, assuming that all conjugated BPA is cleaved in the gastrointestinal tract. We also simulated this exposure scenario. We took average concentrations at steady state as the average concentration over one dosing interval after the system reached steady state. After simulations of a 1-μg/kg/day intravenous (iv) bolus at all ages, we calculated BPA bioavailability (*F*) as


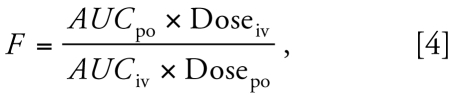


where *AUC* is the area under the simulated plasma concentration time curve for an iv or peroral (po) BPA dose.

### Sensitivity Analysis

We used local sensitivity analysis here to pri-oritize the impact of the input parameters on the outcome of interest: the average steady-state plasma concentration (*C*_avg_ss_). We used its simplest form where an input parameter value (*P*_in_) is changed by 1% and the relative change in outcome is calculated when all other input parameters are fixed:


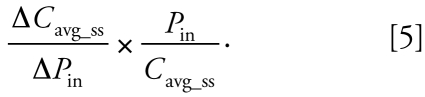


We performed all sensitivity analyses based on a BPA dose of 1 μg/kg/day for an adult. We used sensitivity analyses to assess the variation of both BPA and BPA-Glu organ/tissue volumes (18), organ blood flows (14), hematocrit (1), *f_u_* (1), organ:plasma partition coefficients (15), red blood cell partition coefficient (1), red blood cell permeability (1), organ PS (15), organ-specific PS_rbc_ (18), intrinsic hepatic clearance of BPA (1), and intrinsic kidney clearance of BPA-Glu (1) on *C*_avg_ss_ of both BPA and BPA-Glu.

## Results

### Adult BPA–BPA-Glu coupled model

**Figure 2:**
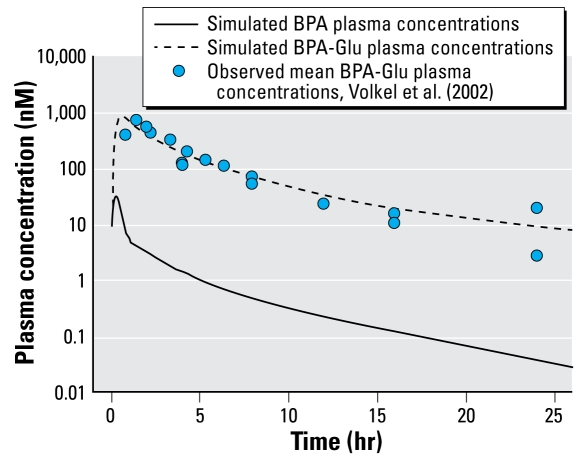
Simulated BPA and BPA-Glu plasma concentration time profile for an adult male after oral administration of BPA. Observed data taken from Volkel et al. (2002).

The plasma CL_BPA_liver_ required to keep the first data point (51 min) below the limit of detection of 10 nM (Volkel et al. 2002) was 21 mL/min/kg. This corresponded to 88% of liver blood flow. The optimized plasma clearance (CL_BPA-Glu_kidney_) for the urinary excretion of BPA-Glu and the BPA-Glu lipophilicity were 1.82 mL/min/kg and −0.05, respectively. CL_BPA-Glu_kidney_ was equal to that derived by Volkel et al. (2002). At 12 and 24 hr, the estimated dose excreted to urine was 83% and 91%, compared with 91% and 94% as observed by Volkel et al. (2002), respectively. Table 4 presents the estimated tissue:plasma partition coefficients for BPA and BPA-Glu for adults and children. Table 5 presents the PS products for adults. Figure 2 presents the optimized plasma concentration time profiles for BPA and BPA-Glu in adults.

### Simulations in children and adults

**Figure 3:**
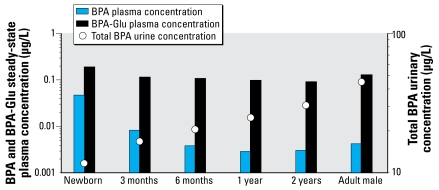
Estimated BPA and BPA-Glu average steady-state plasma concentrations in children and adults after a 1-μg/kg/day oral administration of BPA. Total BPA = BPA + BPA-Glu urinary concentration.

**Figure 4:**
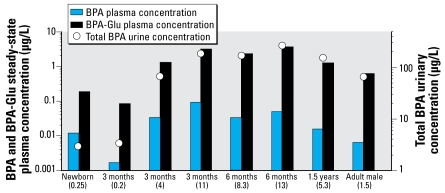
Estimated BPA and BPA-Glu average steady-state plasma concentrations in children and adults after an oral administration of BPA using daily exposures (values in parentheses in μg/kg) taken from EFSA (2006) or, for newborns, estimated from breast milk intake and total BPA breast milk concentration. Total BPA = BPA + BPA-Glu urinary concentration.

At all ages, 100% of the applied BPA dose was modeled as absorbed to the portal vein (*f_a_* = 1), although there was an increase in the efficiency of first-pass metabolism with increasing age. Bioavailability (*F*) in newborns and 3-month-, 6-month-, 1.5-year-olds, and adults was 88%, 48%, 32%, 23%, and 18%. In all simulations, BPA and BPA-Glu steady state was reached within the first 48 hr. Figure 3 presents average plasma concentrations at steady state and urinary concentrations for children and adults after BPA administration of 1 μg/kg/day. Figure 4 presents the average plasma concentrations at steady state of BPA and BPA-Glu as well as the expected urinary concentrations after typical exposure scenarios in children and adults.

### Sensitivity analysis

Values > 1 were of interest in that they identified input parameters that had potential to greatly affect *C*_avg_ss_. No varied parameter in the BPA model produced a value > 1, with respect to BPA average steady-state concentrations, although values to note (> 0.1) were *f_u_* (0.98), hematocrit (0.16), and intrinsic hepatic clearance (0.89). No varied parameter in the BPA model, with respect to BPA-Glu average steady-state concentrations, produced a value greater than 1. No parameter in the BPA-Glu model, with respect to BPA-Glu average steady-state concentrations, produced a value > 1, although a value to note was intrinsic renal clearance (0.80).

## Discussion

Existing PBTK models of BPA and/or BPA-Glu were not appropriate for use in this scaling study. Teeguarden et al. (2005) described a partial PBTK model consisting of five compartments for BPA and a one-compartment model for BPA-Glu. Teeguarden et al. (2005) parameterized this rat model using *in vivo* data from rats after BPA exposure with subsequent scaling to humans. The lumped compartments in the model did not provide an appropriate structure that could account for the relevant physiologic inputs that are required to reliably scale from adult humans to children. This necessitated the use of another model. Shin et al. (2004) adopted a more whole-body PBTK approach and used *in vivo* data from rats for parameterization. Their model focused on BPA, not BPA-Glu. Further, they measured tissue:plasma partition coefficients 3–4 hr after initial exposure, which for a lipophilic compound such as BPA would not be sufficient to reach steady state. The result was a partition coefficient in adipose tissue that was lower than all other organs as well as blood, a result in stark contrast to data from Csanady et al. (2002), who demonstrated in *ex vivo* experiments that the adipose:plasma partition coefficient was two to three times higher than all other tested tissues. Although in rat this value may not be particularly important for describing the kinetics of a lipophilic compound, because only approximately 4% of body weight is adipose tissue, humans have approximately 15–30% of body weight as adipose tissue, so this value is greatly influential. Table 4 presents experimental tissue:plasma partition coefficients from various experimental studies (Csanady et al. 2002; Shin et al. 2004; Yoo et al. 2000). The estimated partition coefficients from the Rodgers and Rowland (2006) and Rodgers et al. (2005a, 2005b) algorithm were within range of the experimental values and deemed reasonable for use in this model. Based on the above limitations in existing BPA models, we used a whole-body PBTK model previously used for scaling drug pharmacokinetics from adults to children (Edginton et al. 2006b). This model has been shown to be physiologically consistent in adults and in children down to newborns. Some examples of this consistency include the following: sum of blood flows = cardiac output; sum of splanchnic organs = portal vein flow; total body water, lipid, and protein is age dependent and consistent with the literature; and addition of blood pool volumes and vascular volume for each organ = total blood content (Edginton et al. 2006b). With the integration of the Rodgers and Rowland (2006) and Rodgers et al. (2005a, 2005b) algorithm for partition coefficient estimation, and the gastrointestinal tract model of Willmann et al. (2003b, 2004), all relevant physiology could be accounted in order to scale BPA and BPA-Glu from adults to children.

Optimization of the BPA-Glu lipophilicity parameter as a means to estimate tissue:plasma partition coefficients from the Rodgers and Rowland (2006) and Rodgers et al. (2005a, 2005b) algorithm resulted in a BPA-Glu distribution volume that was equivalent to that from the Volkel et al. (2002) study (0.43 L/kg). BPA-Glu partition coefficients were, on average, 10 times lower than those of BPA. This is reasonable because glucuronidation and, in general, phase II metabolism aim at increasing water solubility and thus, in the absence of specific binding, results in lower partition coefficients than for the parent compound.

Young children are physiologically different from adults, and this could be considered in the PBTK model that we developed for BPA and BPA-Glu. The activity of many hepatic enzymes is lower at birth, and the activity increase with age occurs at an enzyme-specific rate (Edginton et al. 2006a). In the case of BPA, we considered UGT2B7 the enzyme responsible for BPA hepatic clearance, where newborns had only 5% of the enzymatic activity of the adult liver. This was also responsible for a relative lack of first-pass effect in newborns (*F* = 88%) and subsequently a bioavailability decrease with age. Taken with a distribution change over age (↑ *f_u_*, ↓ *K*_adipose_), the dose-normalized BPA plasma concentrations at steady state were a maximum of 11 times higher than that in adults. As enzyme activity reached that of adults at 1 year of age, the dose-normalized BPA plasma concentrations at this age were lower than in adults because of a higher weight-normalized plasma clearance. This phenomenon has been documented previously (Edginton et al. 2006a). It is of interest that this pediatric PBTK model has been previously used to scale the pharmacokinetics of the UGT2B7 substrate morphine to children with acceptable accuracy (Edginton et al. 2006b). Adult morphine plasma clearance, primarily due to hepatic UGT2B7 activity, is 20.5 mL/min/kg for adults (Edginton et al. 2006a), a clearance similar to that defined for BPA UGT2B7 plasma clearance (21 mL/ min/kg). Thus, the scaling of BPA clearance follows closely with that of morphine.

It is important here to note the limitations of the model with respect to clearance scaling. We generated the BPA plasma clearance in adults based on maintenance of the BPA plasma concentrations that were lower that the limit of detection in the Volkel et al. (2002) study. This value of 21 mL/min/kg represented the lower bound of clearance because we could still meet the criteria given a higher clearance up to a blood flow limitation at around 24 mL/min/kg, with little change in BPA-Glu concentrations (average BPA-Glu concentrations not sensitive to hepatic intrinsic clearance in the BPA model). For the purposes of clearance scaling, our approach to using the lower bound represented the “worst-case scenario” when scaling to children. Scaling clearance for a blood flow limited clearance compound is not possible because intrinsic clearance approaches infinity when blood flow equals blood clearance. In this case, the activity of the enzyme is unknown, and the clearance in the child could reach 100% of hepatic blood flow despite a reduction in intrinsic enzyme activity. Thus, the method we used to generate an adult clearance was the most conservative with respect to pediatric clearance scaling because it represented a worst-case scenario. Further, when clearance scaling to young children, there exists uncertainty when using enzyme activity ontogeny data (Bjorkman 2006) because a period of high interindividual variability is present between the postnatal onset and expression increase of hepatic enzymes (Hines 2007). This has been demonstrated for glucuronidating enzymes (Strassburg et al. 2002) and is expected to be relevant to this discussion regarding BPA. Therefore, the clearance scaling method is built on average *in vitro* activity levels and is not conservative in predicting plasma concentrations in the most developmentally delayed pediatric liver.

We performed a sensitivity analysis to assess the relative importance of the input parameters in affecting the average steady-state BPA concentrations. We performed only a local sensitivity analysis here, so any significant parameter correlations would not be revealed using this method. We found that BPA and BPA-Glu concentrations were not hypersensitive to any one parameter in the model. Of interest to BPA concentrations were the fraction unbound in plasma (0.98) and the hepatic intrinsic clearance of BPA (0.89). For the BPA-Glu concentrations, the parameter renal intrinsic clearance of BPA-Glu (0.80) was of interest. To be confident in our scaled model for children, ensuring the accuracy of these parameters for children is important. We scaled the fraction unbound in plasma from adults based on the method of McNamara and Alcorn (2002), a method demonstrated to be useful for a wide variety of drugs within a varied binding range. This scales the binding fraction based on the age-dependent levels of the binding protein (albumin) in blood. In the absence of experimental data, this algorithm represents the best means of scaling the unbound fraction to children. The hepatic intrinsic clearance, as previously discussed, is not readily discernable from the experimental data currently available. For the purposes of clearance scaling, although UGT2B7 ontogeny has been widely examined (Alcorn and McNamara 2002; de Wildt et al. 1999; Edginton et al. 2006a), the actual adult intrinsic clearance is a difficult parameter to measure given the lack of sensitive analytical techniques for BPA and the borderline blood flow limitation of clearance in the adult. Given the relative importance of this parameter on the outcome of this model, its uncertainty may denigrate the modeling effort to a screening level exercise as opposed to a definitive study and suggests the need to determine adult BPA clearance with certainty. We scaled the renal clearance of BPA-Glu based on the method of Hayton (2000), as modified for children < 2 days of age by Edginton et al. (2006a). This method represents the most widely used renal clearance scaling algorithm (Alcorn and McNamara 2008), and we expect that it accurately predicted renal BPA-Glu clearance in children < 2 years of age.

Volkel et al. (2005) measured plasma concentrations in 19 randomly chosen, unintentionally exposed individuals. Plasma concentrations of total BPA (after glucuronidase treatment) in adults were always below the limit of detection of 0.5 μg/L (Volkel et al. 2005). The average steady-state plasma concentration of total BPA in adults after a typical exposure of 1.5 μg/kg/day was 0.58 μg/L (Figure 4), which suggests that this estimated exposure value is higher than that of the population studied by Volkel et al. (2005). We took typical BPA exposures for adults and children from an EFSA review (2006), although other exposure scenarios have been documented (Lakind and Naiman 2008; NTP 2008). Because only using passive diffusion processes explain the kinetics and because linear BPA and BPA-Glu clearance are assumed, plasma concentrations can be scaled directly with daily exposure dose. This is also the case with urinary concentrations. Using the exposure scenarios of EFSA and assuming conservative migration behavior of BPA from food containers (EFSA 2006), BPA average plasma concentrations at steady state in 3-month-olds (4 μg/kg/day) and 6-month-olds (8.3 μg/ kg/day) were approximately five times greater than those in adults. Breast-fed newborns and 3-month-olds, under typical exposure scenarios, had concentrations 1.8-fold and 0.26-fold, respectively, that in adults (Figure 4).

Because of the relative ease of urine sampling compared with blood sampling, the greatest body of exposure literature is based on urinary concentrations of BPA after sampling of unintentionally exposed individuals. The literature contains little information on children. Based on urinary BPA concentrations, there was a tendency toward higher calculated BPA intakes and urinary concentrations in children and adolescents 6–19 years of age (Lakind and Naiman 2008) compared with adults. We showed higher urinary concentrations in young children than in adults (Figure 4) under typical exposure scenarios, although our urinary concentrations were approximately 25 times higher than those found in adults in both the Lakind and Naiman (2008) and Dekant and Volkel (2008) studies, leading to doubt as to how typical the EFSA exposure scenarios really are. Although Dekant and Volkel (2008) suggested that there is an uncertainty of approximately one order of magnitude when using single spot urine samples to estimate total daily intake by an individual, as was the case in the Lakind and Naiman (2008) and Dekant and Volkel (2008) studies, the EFSA (2006) exposure scenarios are apparently high-end estimates of BPA intake for adults and likely also for young children. Determining actual daily intakes in children < 6 years of age, as recommended by Lakind and Naiman (2008), is important because real exposures in this age group are unknown. Once urinary concentrations are known, back-calculations can be made to estimate daily exposures, and then, using the PBTK model, circulating BPA plasma concentrations may be estimated. This can be of interest when assessing the risks associated with BPA exposure in children. Urinary concentrations at steady state can be used to back-calculate to daily exposures (Lakind and Naiman 2008), although this does not indicate the internal BPA load. Input of the estimated daily exposure into the PBTK model could be used to estimate the circulating BPA concentration and/or tissue concentrations of interest. In this second step, however, uncertainly exists such that there is interplay between plasma concentrations and enzyme activity (for BPA) or urinary clearance (for BPA-Glu) that cannot be directly known simply from a urine sample. Reducing this uncertainty would require direct validation of the PBTK model in young children. This validation would come in the form of blood concentrations of BPA and BPA-Glu in a number of young children. Once acceptance and/or modification of the model is completed, we can extrapolate among ages, doses, and disease states as well as estimate tissue loads with greater confidence.

In summary, we developed a PBTK coupled model system for BPA and its metabolite, BPA-Glu, in adults and scaled to children based on age-dependent physiologic parameters. Because of low UGT2B7 activity, BPA plasma concentrations could be approximately 11 times greater in newborns than in adults exposed to the same weight-normalized dose. An increase in age to 3 months lowers this ratio to 2, although exposure through food can be greater in this age group than in adults (EFSA 2006; NTP 2008). When considering different feeding scenarios, the highest and lowest BPA concentrations differed by a factor of 55. Thus, age-dependent differences may be substantial but cannot be absolutely quantified because of a need for both clearer understanding of the *in situ* hepatic intrinsic BPA clearance in the adult and urinary concentrations in children < 6 years of age to define daily BPA intake. As a worst-case scenario, these findings suggest that the typical intraspecies uncertainty/safety factor of 10 commonly applied to a threshold dose (Pest Management Regulatory Agency 2007) is just sufficiently protective of very young children who have average or above average glucuronidation capacity. EFSA derived a tolerable daily intake of 50 μg (BPA)/kg based on the application of a 100-fold uncertainly factor (10 for interspecies differences and 10 for interindividual differences) to a no observable adverse effect level of 5 mg/kg/day in rats. It is apparent from this modeling study that newborns with less than average glucuronidation capacity may not be adequately considered based on a factor of 10 to account for human variability in BPA toxicokinetics.

## Figures and Tables

**Table 1:** BPA and BPA-Glu compound properties.

Property	BPA	BPA-Glu
Lipophilicity	Log *K*_ow_ = 3.4 (Staples et al. 1998)	−0.05^a^
Molecular weight (g/mol)	228; 244^b^	404; 418^b^
p*K*_a_	9.6, 10.2 (Staples et al. 1998)	Used same as for BPA
Water solubility (mg/L)	120–300 (Staples et al. 1998)	NA
Blood:plasma ratio	1.05^c^	0.83^c^
Plasma protein binding	*K_d_* = 100 nmol/mL (Csanady et al. 2002) *f_u_* = 3.5%	*f_u_* = 95% (deduced from Volkel et al. 2002)
Intestinal permeability (P_int_)	2.8 × 10^−5^; 2.6 × 10^−5^^a^	NA

Abbreviations: NA, not applicable.

a Optimized value using experimental plasma concentration time data from Volkel et al. (2002).

b Relevant for Volkel et al. (2002) simulations because they administered d16-BPA. The first printed value was used for all simulations in children.

c Estimated in PK-Sim. Experimental BPA blood:serum ratio in rats is 1.1 (Shin et al. 2004).

**Table 2:** Parameter values of body weight, body height, and hematocrit as well as organ or tissue volumes (g) and organ blood flows (mL/min) as used in the adult and pediatric PBTK models.

	Age						
Tissue	Newborn	3 months	6 months	1 year	1.5 years	2 years	Adult^a^
Adipose	906/30	1,758/55	2,596/67	3,666/60	3,999/75	4,245/90	14,868/325
Body height (cm)	51	58	65	76	82	88	176
Body weight (kg)	3.5	5.4	7.2	10.0	11.7	13.0	73.0
Bone	452/30	677/44	922/53	1,377/60	1,696/80	1,940/97	11,818/324
Brain	395/180	576/306	754/448	988/700	1,077/898	1,151/1,095	1,508/780
Gonads	1.1/0.3	1.3/0.4	1.5/0.5	1.8/0.6	2/0.8	2/1	40/3
Hematocrit	0.58	0.35	0.36	0.36	0.36	0.36	0.47
Heart	28/24	38/34	48/40	66/48	80/65	90/80	417/260
Kidneys	38/110	55/160	72/192	103/230	126/311	141/378	438/1,325
Large intestine	21/24	30/35	40/42	58/48	72/63	84/76	412/260
Liver	185/39	256/55	325/65	452/78	552/106	627/131	2,357/423
Lung	84/588	119/906	153/1,167	216/1,536	264/2,016	295/245	1,294/6,106
Muscle	968/31	1,311/45	1,628/55	2,215/72	2,767/95	3,287/116	32,338/1,106
Pancreas	9/6	13/9	19/11	28/12	35/16	39/20	190/65
Skin	216/30	281/42	334/49	417/60	483/79	529/95	3,761/325
Small intestine	36/60	52/86	68/103	99/120	125/160	148/194	724/650
Spleen	17/18	25/26	33/32	49/36	60/49	68/60	243/195
Stomach	8/6	12/9	16/10	23/12	29/16	35/19	168/65
Venous blood	38	48	55	68	85	101	691
Arterial blood	38	48	55	68	85	101	691
Portal blood	57	72	83	102	127	151	1,037

Double values are organ weight (g)/organ blood flow (mL/min).

a Volume represents “wet weight” where vascular volume is added to organ volume as given in ICRP (2002).

**Table 3:** Estimated BPA exposure scenarios for infants > 3 months of age taken from the EFSA (2006) report and breast-fed newborn exposure as calculated from Casey et al. (1986) and Ye et al. (2008).

Age	Food/beverages consumed	Exposure/day (μg/kg)	No. of feedings/day
Newborn	Breast-fed only	0.25	8
3 months	Breast-fed only	0.2	8
3 months	Formula fed with polycarbonate bottle	4.0^a^ (11.0^b^)	8
6 months	Formula fed with polycarbonate bottle and commercial foods/beverages	8.3^a^ (13.0^b^)	6
1.5 years	2 kg commercial foods/beverages	5.3	6
Adult	3 kg commercial foods/beverages	1.5	4

a Based on a conservative value for migration of BPA into infant formula (EFSA 2006).

b Based on an upper value for migration of BPA into infant formula (EFSA 2006).

**Table 4:** The ratio of estimated plasma unbound fraction in children (*f_u_*_,child_) to the reported unbound fraction in adults (*f_u_*_,adult_) and the estimated tissue:plasma partition coefficients for BPA and BPA-Glu.

Parameter	BPA^a^	BPA-Glu^a^	Shin et al. (2004)^b^	Yoo et al. (2000)	Csanady et al. (2002)^c^
*f_u_*_,child_/*f_u_*_,adult_ (age)					
Term neonate	1.29	1.01			
3 months	1.28	1.01			
6 months	1.27	1.01			
1 year	1.25	1.01			
2 years	1.23	1.01			
Adipose (age)					
Term neonate	3.8	0.59			
3 months	5.6	0.43			
6 months	5.7	0.42			
1 year	5.8	0.41			
2 years	5.9	0.40			
30 years	8.3	0.18	0.7 ± 0.6		3.31 ± 0.17
Bone	1.7	0.42			
Brain	3.6	0.75	4.4 ± 0.6	0.75	1.06 ± 0.09
Gonads	0.79	0.83	3.4 ± 0.5	2.8	
Heart	1.6	0.57	3.4 ± 1.0	3.3	
Kidneys	1.8	0.68	4.4 ± 0.7	4.9	1.35 ± 0.17
Large intestine	3.8	0.75		4.1	
Small intestine	3.8	0.75	45.96 ± 3.2		
Stomach	3.8	0.75	4.6 ± 0.9	4.1	
Liver	1.9	0.65	5.7 ± 2.2	2.0	1.46 ± 0.38
Lung	2.4	0.58	5.5 ± 0.5	5.7	
Muscle	2.2	0.74	0.8 ± 0.1		1.35 ± 0.31
Pancreas	3.9	0.65			
Red blood cells	1.1	0.65			
Skin	5.7	0.69			
Spleen	1.0	0.55	2.8 ± 0.8	2.9	

a The age-specific partition coefficient is the product of the ratio of unbound fractions and the partition coefficient.

b *Ex vivo* exposure (mean ± SD) of 4–5 g of cut-up human tissue to BPA over 6 hr in a flask; *n* = 3–4.

c BPA exposure to rats via multiple intravenous infusions, with tissues taken at around 3.5 hr (*n* = 2 rats) and 4.5 hr (*n* = 2 rats) after dose initiation.

**Table 5:** Permeability × surface area (PS) products (L/min) for BPA and BPA-Glu for adults.

	BPA		BPA-Glu	
Parameter	PS_organ_	PS_rbc_organ_	PS_organ_	PS_rbc_organ_
Venous/arterial		1,675		0.018
Adipose	4,725	648	0.051	6.9 × 10^−3^
Bone	4,766	973	0.051	0.010
Brain	0.51	142	5.4 × 10^−6^	1.5 × 10^−3^
Gonads	82	5.7	8.9 × 10^−4^	5.7 × 10^−5^
Heart	3,123	142	0.033	1.5 × 10^−3^
Kidneys	14,427	244	1.2	2.6 × 10^−3^
Large intestine	5,145	24	0.83	2.6 × 10^−3^
Liver	25,389	971	0.27	0.010
Lung	59	1,819	6.3 × 10^−4^	0.019
Muscle	2,752	1,959	0.029	0.020
Pancreas	16,529	92	2.3	9.9 × 10^−4^
Portal vein blood		2,513		0.027
Skin	17	419	1.9 × 10^−4^	4.5 × 10^−3^
Small intestine	18,116	42	1.3	4.5 × 10^−4^
Spleen	60,468	195	2.8	2.1 × 10^−3^
Stomach	24,035	13	2.4	1.4 × 10^−4^

rbc, red blood cells.
